# Surface Reconstruction through Poisson Disk Sampling

**DOI:** 10.1371/journal.pone.0120151

**Published:** 2015-04-27

**Authors:** Wenguang Hou, Zekai Xu, Nannan Qin, Dongping Xiong, Mingyue Ding

**Affiliations:** 1 Department of Biomedical Engineering, Huazhong University of Science and Technology, Wuhan, China; 2 Electric Power Planning & Engineering Institute Beijing North-Star Co.,Ltd., 65-2 Ande Road, Beijing, China; Xiamen University, CHINA

## Abstract

This paper intends to generate the approximate Voronoi diagram in the geodesic metric for some unbiased samples selected from original points. The mesh model of seeds is then constructed on basis of the Voronoi diagram. Rather than constructing the Voronoi diagram for all original points, the proposed strategy is to run around the obstacle that the geodesic distances among neighboring points are sensitive to nearest neighbor definition. It is obvious that the reconstructed model is the level of detail of original points. Hence, our main motivation is to deal with the redundant scattered points. In implementation, Poisson disk sampling is taken to select seeds and helps to produce the Voronoi diagram. Adaptive reconstructions can be achieved by slightly changing the uniform strategy in selecting seeds. Behaviors of this method are investigated and accuracy evaluations are done. Experimental results show the proposed method is reliable and effective.

## Introduction

The applications of inferring a surface from the scattered points are wide ranging such as finite element analysis, reverse engineering, 3D games and virtual reality. Studies have being constantly focused on this topic to improve the algorithm’s robustness to noise and reduce the computation complex. The more important is to improve the reliability and accuracy of the reconstructions. Meanwhile, the scenarios of scattered points are continuously emerged with the development of hardware equipment. Then, surface reconstruction remains an important and hot topic in related fields [1][2]. Now, it is common that the scattered points are usually dense thanking to the advanced laser-scanner, computer vision and medical imaging devices. For these dense and redundant scattered points, in most cases, only a small portion of original points will be resided in the final model. A direct way for dense points reconstruction is to simplify the original data and then construct the mesh model for the simplified points. However, it isn’t always feasible due to constructing a mesh model with a certain number of vertexes on basis of some more points is reliable. Hence, the traditional methods are to construct the meshes for original points and then perform remeshing to accomplish simplification and optimization for the initial meshes [3][4][5][6]. These traditional methods generally consume much memory and computation especially when the original points are in great amount. In view of this fact, this paper intends to investigate a new method to merge the process of initial meshing and mesh optimization into one step. Moreover, the proposed method should be memory and computation economical. To attach it, some unbiased seeds will be selected from the original data to establish the mesh model which is the Level of Detail (LOD) of original points. A classic sampling method, Poisson disk sampling, serves for selecting seeds and the original points located in different disks are used to judge whether two seeds should be connected in the final meshes. Rather than designing an algorithm for robustness to noise and un-uniformity, this paper is to provide a simple, high speed and local computation to achieve surface reconstruction by exploiting the redundant information.

## Related Works Analysis

Surface reconstruction from unorganized points is really a heavily studied field. Many excellent approaches have been designed to deal with it including marching cubes [7], Poisson surface reconstruction [8][9], curvature-adaptive implicit surface [2] [10], Voronoi-based computational geometry [11] [12] [13] [14] and moving least squares [15]. According to the situations whether the input points appear as vertices of the output meshes, we simply divide them into two kinds, implicit surface approach and Voronoi-based method.

Hoppe [7] [16] [17] early brought this problem to the attention of computer graphic community. In his dissertation seminar, marching cubes for surface reconstruction was completed in which a tangent plane at each sample was estimated using the *k*-nearest neighbors, and then the distance to the plane was used to compute a signed distance function. The zero set of this function was contoured by a continuous piecewise linear surface. Poisson surface reconstruction considered the resurfacing as a spatial Poisson problem to seek an indicator function that best agrees with a set of noisy, non-uniform observations [9]. The solution to the Poisson problem was transformed into a well conditioned sparse linear system by introducing a locally supported basis function. Finally, iso-surface was extracted from the indicator function. Later, parallel Poisson surface reconstruction was introduced to accelerate the reconstruction [8]. Moving least-squares classified the regions of a point cloud into multiple outlier-free smooth areas using the forward-search paradigm [15]. Then, it projected these points on a locally smooth region rather than a surface that was smooth everywhere, and a piecewise smooth surface was subsequently defined. Curvature-adaptive implicit surface reconstruction traced the zero crossings of a signed field obtained from the sum of first-derivative anisotropic Gaussians centered at each point [2]. Its key characteristic is the ability to smooth more along edges than across them, thereby preserving shape details while smoothing noise. However, some parameters and functions should be defined by users to balance the royal to original samples and the robustness to noise.

Voronoi-based resurfacing method can be traced into the curve reconstruction from scattered 2D points. In [18], the crust idea was developed through performing the Medial Axis Transform (MAT). Afterwards, Amenta et al. [11] [19] extended the crust principle to 2D manifold as the power crust. It estimated the MAT of object to be reconstructed from samples and then applied an inverse transform to the MAT to produce a piecewise-linear hollow object representation. Another well-known Voronoi-based geometry reconstruction method is the Alpha shape [13] [14]. It firstly performs 3D triangulation for original points. Then, each simplex (edge, face, point) may belong to the Alpha shape if the radius of its circumsphere is less than the value of Alpha. It is difficult and sometimes impossible to choose the Alpha value to comprise hole filling and loss of details. Edelsbrunner et al. [13] are continuing to improve the Alpha shape by assigning a weight with interpretation that a large (small) weight favors (discourages) connections to neighboring points. In addition, some researchers try to perform surface meshing utilizing 2D Delaunay triangulation algorithm [20] [21], in which, the main difficulty lies in how to mix together neighboring meshes.

The Voronoi-based methods are more direct than the implicit surface approaches in that they retain the input points, as given, to appear as vertexes of the output triangle meshes. With advanced development in Voronoi computation such as in CGAL [22], Voronoi-based methods are greatly accelerated. For the implicit surface methods, the vertexes are not necessarily the original ones as well as not all samples are necessarily the vertexes of reconstructed meshes. The vertexes in the final meshes are actually interpolated by a smooth function, which makes them be insensitive to noise. In applications, these methods can obtain fine results for uniformly distributed samples and their alterations tried to fit to more common scenarios that are existence of noise and non uniformity [23]. And to some extent, these methods are mathematically complicated such as fast Voronoi diagram generation and Poisson equation computation. Furthermore, they generally perform two steps (meshing and optimization) to achieve reconstruction. Then, it is of practical significance to design a new resurfacing method which should be conceptual simple and can synchronously achieve meshing and remeshing. By considering the fact that the redundant data are common, our method is formed on basis of Poisson disk sampling [24]. It belongs to the Voronoi-based resurfacing method.

## Methods

The proposed method is to select some seeds from original points, generate the approximate Voronoi diagram of the seeds taking the geodesic distances as basis and then derive the corresponding triangle meshes. Finally, small holes will be filled according to the constraint delaunay rule [25].

### 1D manifold case

**Fig 1:**
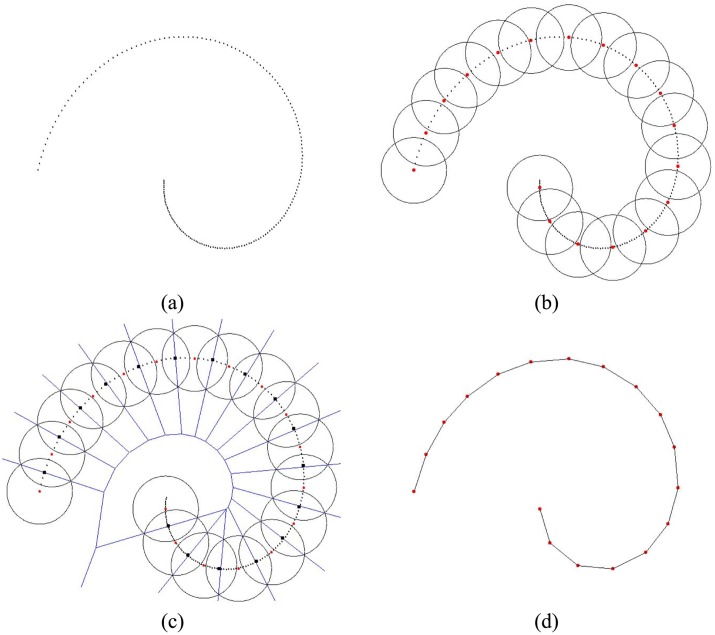
Select the seeds, find neighbor seeds and connect them to reconstruct a curve.

Fig 1(a) shows the 2D scattered point set sampled from a smooth curve. It is dense and redundant. Some seeds are selected through Poisson disk sampling as the red points in Fig 1(b). The areas surrounded by black circles denote the related disks. The distances between any two seeds are more than disk’s radius and no more seeds can be selected from original points. Meanwhile, some original points may have same distances to two seeds as the black rectangles in Fig 1(c). We then can consider that two seeds are in neighbors if there is a black rectangle on their disks’ common region. According to the computational geometry, these black rectangles should be located on the Voronoi edges of these seeds as the blue lines. At last, two neighbor seeds are connected and the reconstruction is in Fig 1(d). It should be mentioned that the distance between any two points are estimated using the geodesic distance rather than Euclidean distance since geodesics builds the metric for scattered points along the curve to be reconstructed.

### 2D manifold reconstruction

**Fig 2:**
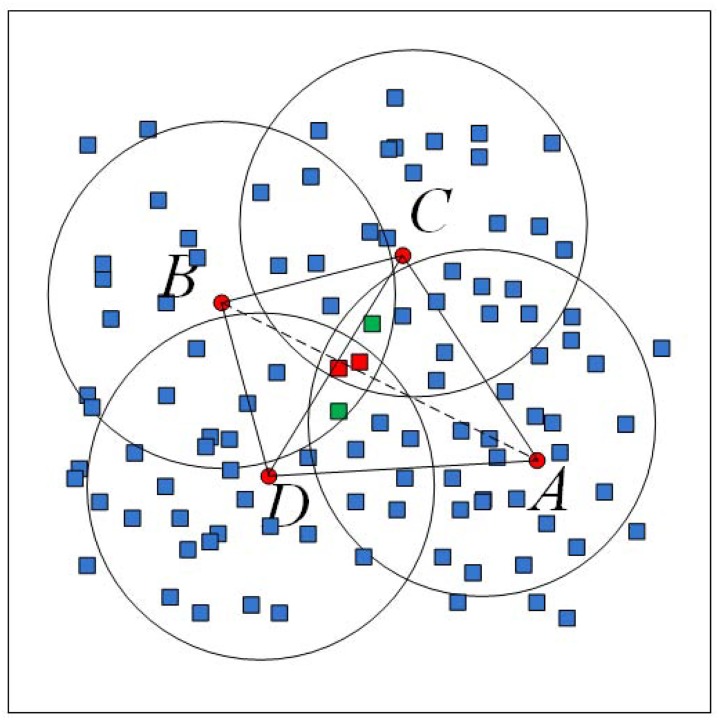
Select seeds, find the points near voronoi edges and construct meshes in 2D manifold case.

Fig 2 shows our method in 2D manifold case, in which, red dots denote the seeds and rectangles represent other original points. Red rectangles appear in the disks of *A*, *B*, *C* and *D*. Meanwhile, two green ones are located in the overlapped region of *A* and *B*. It means that the geodesic distances from each of these 4 rectangles to *A* and *B* are closely equal. Moreover, two red rectangles are farther from *A* and *B* than to *C* and *D*. I. e., the geodesic distance between *C* and *D* is less than that of *A* and *B*. Then, a edge between *C* and *D* will be appeared in the final meshes. Here, the difference between 1D and 2D manifold reconstruction can be found. In 1D case, two seeds should be connected if there is a point in their disks’ overlapped region. Yet, a new constraint should be assigned in 2D case that is the common points should be far from other seeds.

Our method is to select some seeds from the original data and connect the neighbor seeds by judging whether two seeds’ disks are overlapped and the points in their common regions are far from other seeds. These common points should be approximately located on the Voronoi edge of the related two seeds. After sampling and finding all points satisfying above, the approximate Voronoi diagram of seeds is actually generated. Its dual mesh is the reconstruction. Due to there may be fewer original points rightly located on the Voronoi edges, we usually set a threshold for geodesic distances’ differences (tenth of ridus) to search the points near Voronoi edges.

### Uniform and adaptive reconstruction

**Fig 3:**
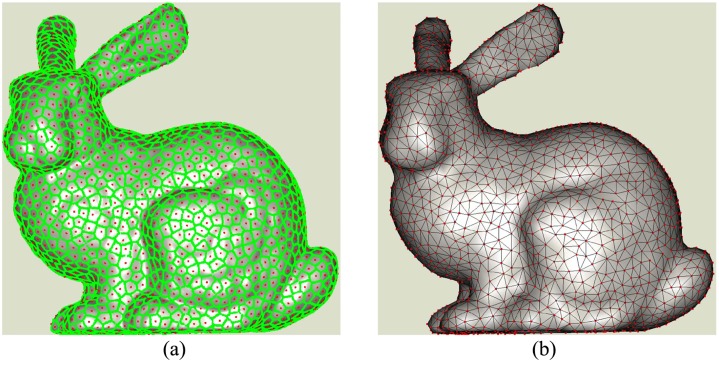
Voronoi diagram of Poisson disk samples.

Uniform reconstruction can be achieved by setting disks’ size be same in selecting seeds. Due to we intend to choose the seeds and find the points located on the Voronoi edges synchronously, the classic dart throwing [26] [27] is introduced here. It randomly selects a seed from original data and search points covered within the disk centered at the selected seed. I. e., the geodesic distances from the seed to those points are less than or equal to disk’s radius. All points covered in the disk will not be selected to guarantee that the geodesic distance between any two seeds is more than the radius [28]. The sampling process is repeated until no seed can be selected. For an point, if its geodesic distances to two seeds are less than or equal to disk’s radius, it must be located in the common region of two seeds’ disks. Further judgement according to subsection 3.2 will be done to generate the approximate Voronoi diagram. For Buddy, the Voronoi diagram is denoted as the green points in Fig 3(a) and its dual meshes are shown in Fig 3(b). The Voronoi diagram and the meshes are almost uniformly distributed. It makes the resolution of reconstruction be almost the same everywhere notwithstanding the densities’ distribution of original points. It is similar as the 1D case in Fig 1(d).

**Fig 4:**
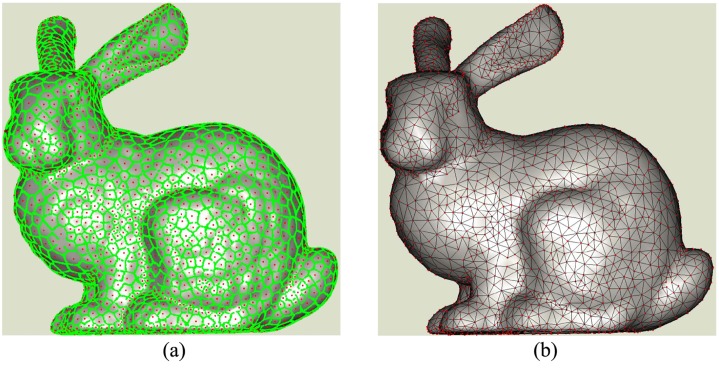
Adaptive reconstruction by Poisson disk sampling.

In some applications, adaptive reconstruction is preferred which means that seeds and meshes are dense in uneven regions and sparse in smooth areas. To make adaptive reconstruction, the disks in rugged regions should cover smaller areas than those of seeds in smooth areas, which results in that the adjacent Voronoi sub-diagrams have different sizes as shown in Fig 4(a). The corresponding adaptive meshes is shown in Fig 4(b). To make Poisson disks in different regions cover areas with different sizes, geodesic distance measurement among points is replaced by the weighted one. It is obvious that the weighted geodesic distance of two points in uneven region should be increased much more than that when these two points are located in smooth areas. This will make relatively more seeds be located in rugged regions after Poisson disk sampling. In the experiments of this paper, we adopt a simple way this is to multiple the original geodesic distance with a exponential function to obtain the weighted one [29]. It is just to illustrate that our method can obtain adaptive reconstruction when the weighted geodesic distance is applied. Sophisticated weight estimation methods will not be discussed.

### Method Analysis

To some extent, the proposed method is similar to the idea of [30] which selects (generates) some seeds from original data and establishes a spherical cover centered at each seed on 2D manifold surface. It then derives mesh model on basis of these spherical cover. The main difference between the proposed method and [30] lies in how to judge the neighbor relationship among seeds. The proposed method actually constructs the approximate Voronoi diagram for the seeds. Yet, Ohtake [30] computed the intersections among different circles of the seeds. It may lead to overlapped and repeated meshes in the reconstruction. By comparison, our meshes are all manifold. Another main difference is that we find the points located in the disk of each seed through geodesic distance computation rather than projecting all points in the tangent plane of each seed. Then, normal computation is unnecessary in our uniform reconstruction.

The second issue is that we generate the Voronoi diagram of seeds rather than all original points. It is due to the error of geodesic distance estimation especially for neighboring points. As widely accepted, geodesic distance between two close points is estimated using their Euclidean distance and geodesic distance between two arbitrary points is approximated by adding up all geodesic distances of close points along the shortest path connecting them [31]. Then, the geodesic distance between two far points will have relatively less error in different definitions of nearest neighbors [1] [32]. I. e., construction of the Voronoi diagram for all original points isn’t feasible. Yet, it is possible for part of them located far away from each other. As a classic problem, geodesic distance computation can be found in [31].

## The logic flowchart

The logic flowchart of proposed method is as follows.

1. Set a radius for Poisson disk sampling and find the neighbors for each point.
2. Randomly select a seed from the original points.
3. Take this seed as source and find points located in its disk using fast marching algorithm [31]. (The geodesic distances from these points to the source are less than Poisson disk’s radius).
4. Mark these points in above step to represent that they are located in the disk of selected seed and will never be sampled as seeds.
5. Return back to step 2 and repeat the process until all points are covered by the disks.
6. Search points which are on or near the edges of Voronoi diagram. Each point should satisfy three conditions: it is located in two seeds’ disks; the difference between the geodesic distances from it to two seeds is less than a threshold (eg. tenth of radius) and it is far away from other seeds.
7. Judge if two seeds are adjacent by identifying whether there are points on or near the Voronoi edge between them.
8. Record the adjacent seeds and construct the corresponding triangle meshes and fill the holes.

Finding *k* nearest neighbors is in *O*(*NLogN*) computation, *N* is the number of original points. Searching points within the disk of each seed consumes *O*((*N*/*s*)^2^) computation using fast marching, *s* is the number of seeds. Then, computation in finding all seeds and the corresponding disks is in *O*(*N* ^2^/*s*) complexity. Voronoi diagram generation needs *O*(*N*) computation. Complexity of constructing the triangle meshes from adjacent edges is about *O*(*N*). *N*/*s* is close to *LogN*. Then, the computation complexity of surface reconstruction through Poisson disk sampling is *O*(*NLogN*).

## Results

The strategy of *k* nearest neighbor is adopted to find the neighbors and ANN library [33] is used to fulfill this task. Sampling interval among original points can be computed by averaging the distances from each point to its neighbors. The original sampling interval will be taken to determine disk’s radius and judge whether a hole in the final meshes should be filled. Some familiar point sets [34] are used to test. Moreover, reconstructions have also been done using other methods including Alpha shape in the software Geomagic [35] and Poisson surface reconstruction packed in the MeshLab [36].

### Uniform and adaptive reconstruction

**Fig 5:**
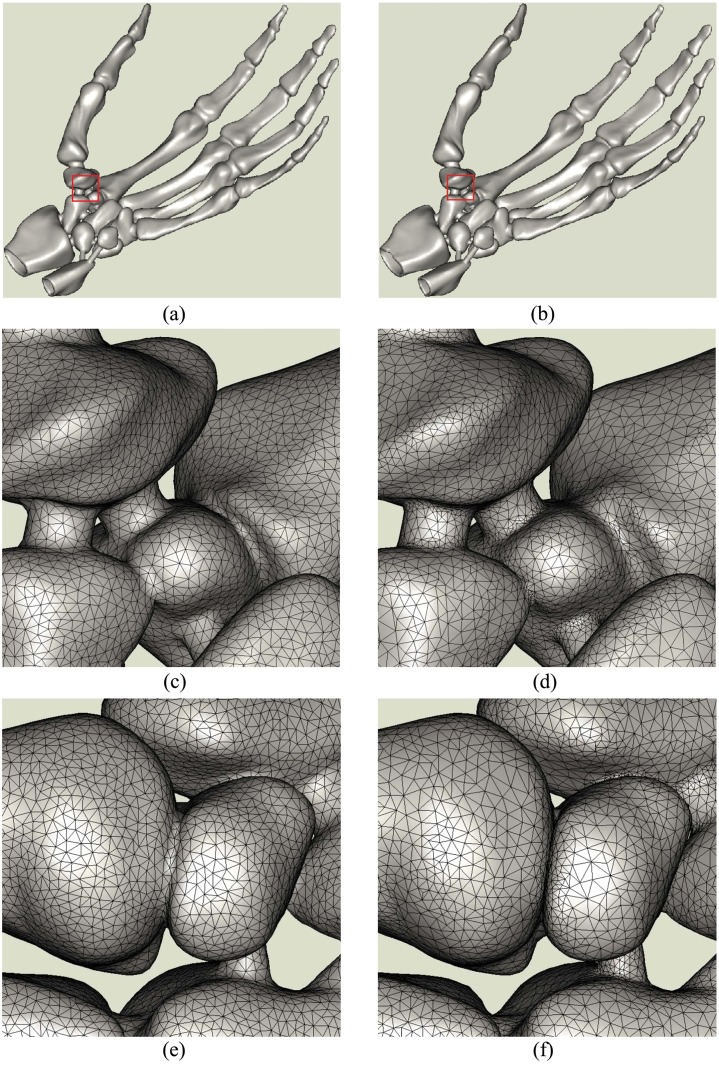
Skeleton Hand’s reconstructions by Poisson disk sampling method. (a), (b) are the uniform and adaptive reconstructions. (c) is the enlarged region of (a) marked with red window, (e)is the back side of (c). (d),(f) are the corresponding images of (b).

The first experiment is to reconstruct a *Skeleton Hand*. The number of points we used is about 3 millions. Uniform and adaptive reconstructions are then carried out. In uniform test, the value *k* in computing neighbors is 30, the disk’s radius is 3 times of the original sampling interval. Fig 5(a), 5(c) and 5(e) show the uniform reconstructions, in which, the triangle meshes are uniformly distributed. The adaptive reconstructions are shown in Fig 5(b), 5(d) and 5(f), in which, more points are located in uneven regions and relatively fewer points appear in smooth parts. In adaptive reconstruction, we set coefficient be 0.25 in computing weighted geodesic distance [29]. Accordingly, the disk’s radius is 3 times of the average weighted geodesic distance among original points in definition of 30 nearest neighbors. The whole shape of the hand is finely restored in these two tests. The superiority of adaptive reconstruction to the uniform one can be clearly found in the enlarged regions in Fig 5(c)–5(f). Therefore, we only list the adaptive reconstructions in later experiments. The remaining points in Fig 5(a) and 5(b) are about 55,000 and the meshes are 111,400. The numbers of seeds and meshes may have some slight differences in different tests due to randomly sampling.

### Other tests on redundant data sets

**Fig 6:**
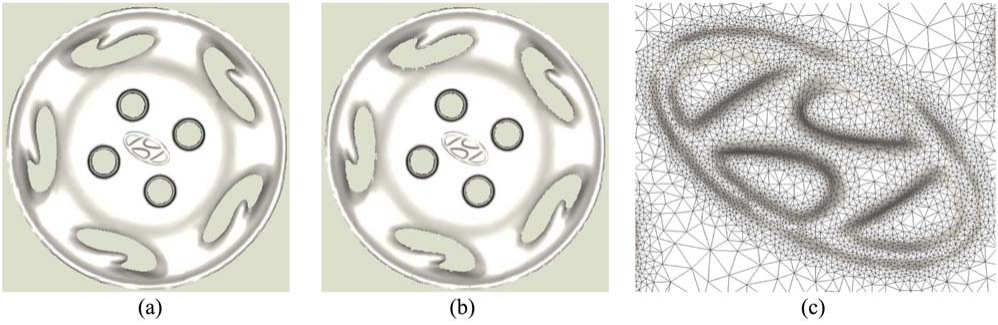
Surface reconstruction of car wheel.

The original number of points in *Car Wheel* is 2 million and the model reconstructed using Alpha shape is shown in Fig 6(a). Our adaptive result is Fig 6(b). There are about 35,000 points and 71,000 meshes. The enlarged region of our result is shown in Fig 6(c). From it, we can see that the distribution of points and meshes compromises the uniformity and adaptivity. The whole shape is adaptive and the local distribution of meshes is uniform. This way effectively saves points in smooth parts and retains the details in uneven regions. Whereas, some defects are existed in the edges due to sampling ratio is a bit low.

**Fig 7:**
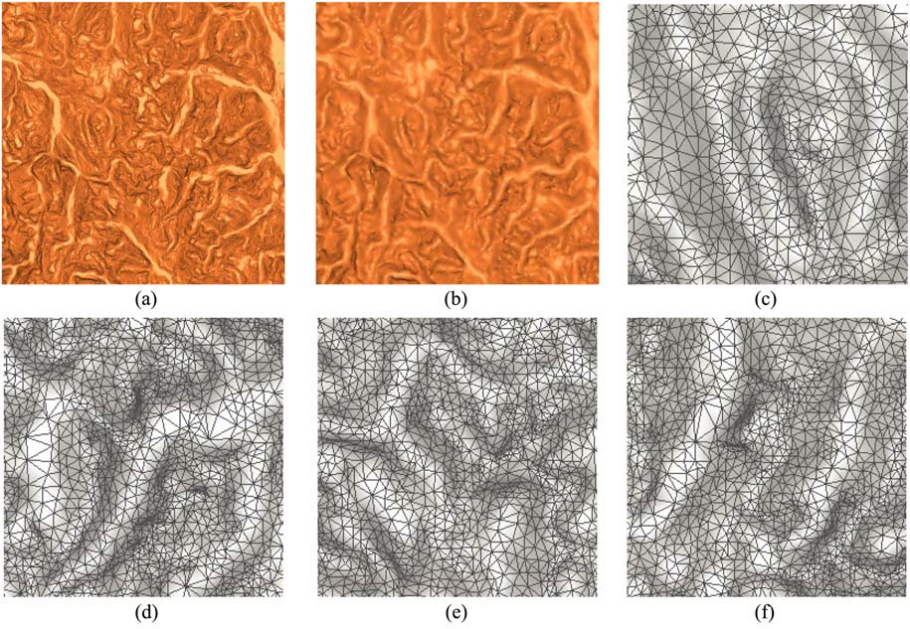
Surface reconstruction models of DEM.

In the field of cartography, mesh is widely taken to describe the Digital Elevation Model (DEM). Generally, 2D Delaunay partition is taken to generate the meshes for the topographical points. In this test, we apply our method to the DEM triangulation. The resolution of original data is very high, which contains more than 2 million points. Fig 7(a) is the mesh model constructed according to 2D Delaunay. Our adaptive one is shown in Fig 7(b) with 22,000 points and 44,000 meshes. It can be found that the resolution of Fig 7(b) is lower than that of Fig 7(a). However, the main details of original data are effectively remained. The enlarged regions of Fig 7(b) are shown in Fig 7(c)–7(f). The distributions of points and meshes rightly reflect the terrain variation that meshes are dense in rugged parts and sparse in flat regions. Although 2D Delaunay partition can obtain relatively good results for DEM in most of situations, yet it has some limitations when cliffs are existed. Thereby, our method should be attractive in applications of DEM mesh partition.

**Fig 8:**
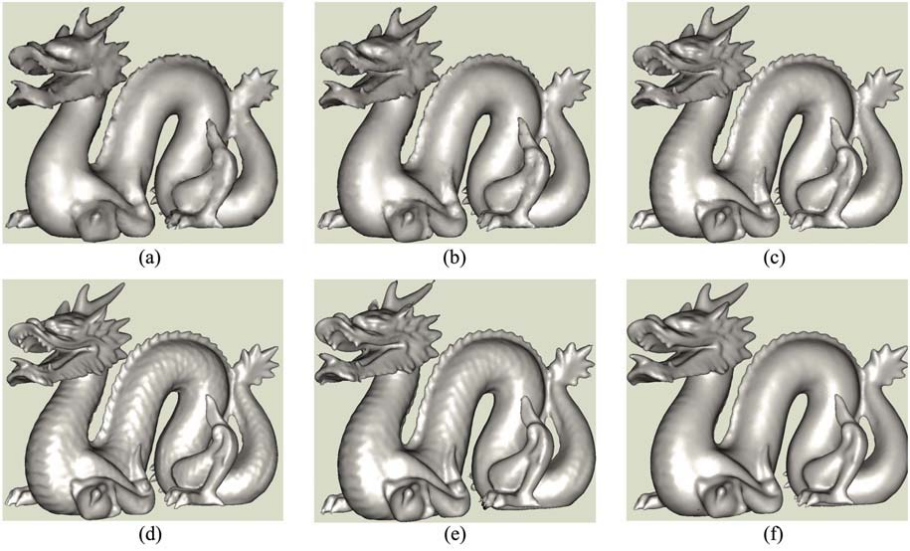
Dragon’s mesh models. (a),(b),(c),(d) are the models reconstructed using our method in different parameters, (e), (f) are the reconstructions by alpha shape and Poisson surface for the seeds in (d).

Fig 8 shows the reconstructions of the Dragon which contains about 2 million original points. For Fig 8(a)–8(d), the numbers of seeds are 4,800, 8,500, 18,000 and 64,000 obtained by adjusting disk’s radius. The parameter *k* in finding the neighbors is 15. The coefficient is 0.3 in determining the weight in weighted geodesic distance computation [29]. The resolution of mesh model increases when the seeds become denser. However, the whole model can still be reasonably recognized when the number of points is 4,800. At the same time, we perform surface reconstructions for the seeds in Fig 8(d) using Alpha shape and Poisson surface reconstruction. Their results are illustrated in 8(e) and 8(f) correspondingly. Visually, the resolution of 8(f) is lower than those of ours, Alpha shape, and the result in [9]. This is mainly due to the points in Poisson surface reconstruction is much less than that in [9]. But overall, three methods can obtain fine results.

### Accuracy evaluation

**Fig 9:**
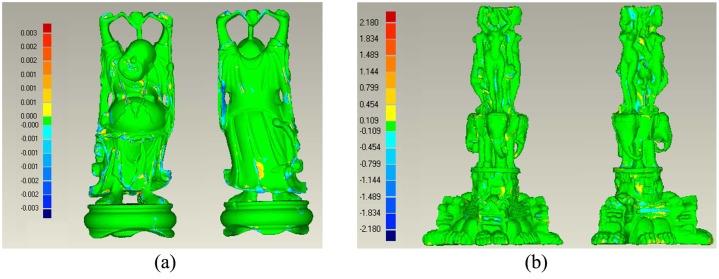
Comparisons between our reconstruction and the “standard model”.

Our reconstruction is the LOD of original data, which means the accuracy of the reconstructed model can’t achieve the same level as others. On another hand, the proposed method will still be preferred when the accuracy reduction can be acceptable. So, this section intends to estimate the accuracy of our reconstructions. We firstly generate the mesh model for original data using the Alpha shape. The model is taken as the ground truth of the manifold to be reconstructed. Here, the Alpha shape is only a example to generate the “standard” model. Other surface reconstruction such as Power Crust, Marching cubes can also be used. Then, we reconstruct the adaptive mesh models for the test data using our method. After that, the difference between our reconstructions and the “standard” model is computed using the function of “3D compare” in software of Geomagic. Fig 9 shows the results after comparison. The numbers of original points are 536,000, 2243,000 in Fig 9(a) and 9(b). The remaining numbers are about 40,000 and 80,000 respectively. From them, we can see that most of our reconstructions highly conforms to the “standard” model and the deviation is relatively less. The average deviations of those two data sets are 0.0004 and 0.5531. The diagonal lengths of these objects are about 0.214 and 504.7. Then, their relative errors are 1/500 and 1/900. Fig 9(b) obtains higher accuracy due to its original data is intensely redundant. In all, the proposed method reconstruct the mesh model at cost of accuracy, however, the amount of loss is limited when the original data are redundant.

**Fig 10:**
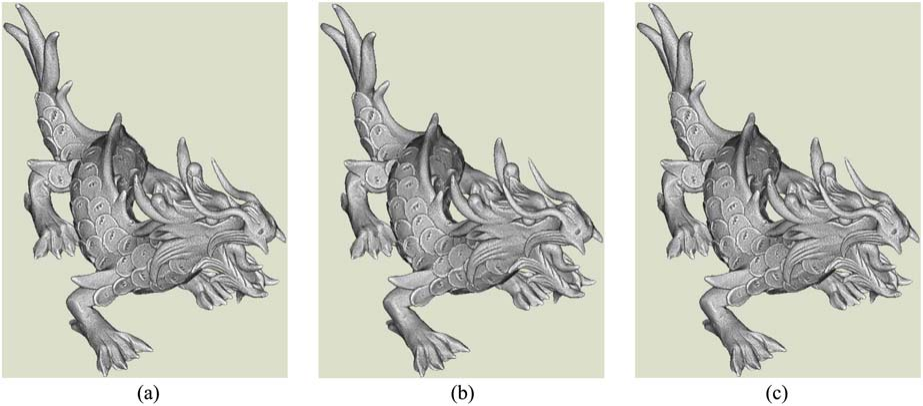
Comparison between our reconstruction and Meshlab’s simplification. (a) is the original dense mesh model, (b) is our reconstruction without referencing initial meshes, (c) is the simplified mesh model using the software of Meshlab. In the process of simplification, the original mesh model is taken as a basis. The differences among three mesh models is minor. It means that the redundant data is sometimes unnecessary. Then, our reconstruction strategy for the redundant points is motivated.

Subsequently, we will compare our result with other LOD models obtained by a calssic method. The tested data is a dense and complex mesh model [34], which contains about 3 millions vertexes and 7 million meshes shown in Fig 10(a). Firstly, the proposed method is utilized to construct the meshes by considering the original vertexes as scattered points. Fig 10(b) shows our reconstruction with 212,524 remaining vertexes. The main parameters in reconstruction are: nearest neighbor number is 15, the Poisson disk radius is 2.3 times of the average distance among original vertexes and the curvature coefficient in weighted geodesic distance is 0.1. Meanwhile, we obtain a simplified model from the original mesh model according to the function of “Quadric Edge Collapse Decimation” in the software Meshlab. Fig 10(c) shows the result with 212,524 vertexes. In the process of simplification using Meshlab, default parameters are used. Visually, our reconstruction and the simplified model are all close to the original one. Moreover, the difference between ours and Meshlab’s is minor. It should be pointed out that the original meshes are not considered in our reconstruction. Yet, Meshlab simplification takes original mesh model as basis. It actually performs simplification and optimization for initial meshes.

**Fig 11:**
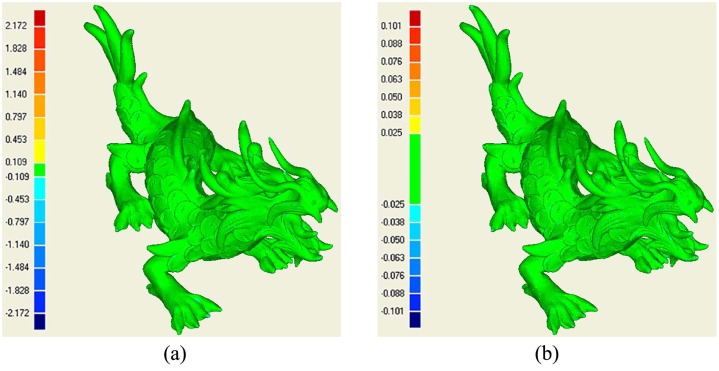
The accuracy estimation by comparing the LOD models with the original one. (a) is difference between our reconstruction and the original dense mesh model and (b) shows the difference between original one and the simplified model.

After then, we estimate their accuracies by comparing them with the original dense mesh model. The “3D compare” in the software of Geomagic is still used to do comparison. Fig 11(a) is the comparison between our reconstruction and the original mesh model. Fig 11(b) shows the corresponding comparison of the simplified model obtained by Meshlab. Our absolute standard error is 0.027 and the value of simplified model is 0.011. Our reconstruction obtains relatively lower accuracy though the difference isn’t very obvious. This result can be comprehended due to the simplified result is obtained by relying on the original mesh model. Yet, our method only considers the dense vertexes as scattered points. On another hand, this experiment effectively demonstrates that our LOD reconstruction is reliable in accuracy. At the same time, our method should be further strengthened to preserve more feature vertexes in the remaining mesh models to improve the accuracy of reconstructions.

## Conclusion

This paper proposes a novel surface reconstruction method that intends to compute the approximate Voronoi diagram for some seeds selected by Poisson disk sampling. It mainly aims to generate the triangle meshes for scattered data containing redundant points. The merits are that the method is conceptually simple and can be easily implemented. Moreover, normal orientation isn’t required and the normal computation can even be ignored in uniform reconstruction. Lots of experiments demonstrate its effectiveness. The main limitation is that it doesn’t fit to sparse point sets. To overcome this obstacle, a basic idea is to unfold the points in each Voronoi region onto a plane and then compute the meshes for these 2D points on basis of constraint Delaunay rule, which actually performs 3D surface reconstruction from coarse to fine. The final meshes will be constructed by all original points. This strategy is attractive and should be our future researches.
